# Model systems for studying trophoblast differentiation from human pluripotent stem cells

**DOI:** 10.1007/s00441-012-1371-2

**Published:** 2012-03-17

**Authors:** Toshihiko Ezashi, Bhanu Prakash V. L. Telugu, R. Michael Roberts

**Affiliations:** 1 Division of Animal Sciences & Bond Life Sciences Center, University of Missouri-Columbia, Columbia, MO 65211 USA; 2 Department of Animal and Avian Sciences, College Park, MD 20742 & Animal Biosciences and Biotechnology Laboratory, ANRI, ARS, USDA, University of Maryland, Beltsville, MD 20705 USA; 3 240b Bond Life Sciences Center, 1201 E. Rollins Street, Columbia, MO 65211-7310 USA

**Keywords:** Pluripotent, Embryonic stem cell, Placenta, Trophoblast differentiation, BMP4

## Abstract

This review focuses on a now well-established model for generating cells of the trophoblast (TB) lineage by treating human embryonic stem cells (ESC) and induced pluripotent stem cells (iPSC) with the growth factor BMP4. We first discuss the opposing roles of FGF2 and BMP4 in directing TB formation and the need to exclude the former from the growth medium to minimize the co-induction of mesoderm and endoderm. Under these conditions, there is up-regulation of several transcription factors implicated in TB lineage emergence within 3 h of BMP4 exposure and, over a period of days and especially under a high O_2_ gas atmosphere, gradual appearance of cell types carrying markers for more differentiated TB cell types, including extravillous TB and syncytioTB. We describe the potential value of including low molecular weight pharmaceutical agents that block activin A (INHBA) and FGF2 signaling to support BMP4-directed differentiation. We contend that the weight of available evidence supports the contention that BMP4 converts human ESC and iPSC of the so-called epiblast type unidirectionally to TB. We also consider the argument that BMP4 treatment of human ESC in the absence of exogenous FGF2 leads only to the emergence of mesoderm derivatives to be seriously flawed. Instead, we propose that, when signaling networks supporting pluripotency ESC or iPSC become unsustainable and when specification towards extra-embryonic mesoderm and endoderm are rendered inoperative, TB emerges as a major default state to pluripotency.

## Introduction

A placenta originating from the outer cells (trophectoderm) of the early blastocyst is the hallmark of mammals. In eutherians, unlike the marsupials, whose placenta is short-lived, the fetus is nurtured in utero for an extended period of time and the placenta becomes well developed and involved in a multitude of processes essential to maintain the pregnancy until the offspring are born. These functions include transport of nutrients, exchange of gases, production of both steroid and polypeptide hormones, structural support within the womb, immunological protection and acting as a physiological buffer between the mother and the fetus; yet, despite these commonalities, the placenta is arguably the most diverse of mammalian organs, showing bewildering variation in organization and gross morphology (Enders and Blankenship 1999; Enders and Carter 2006). For example, within the primate order, placental types range from those that are highly invasive, as seen in the human, to those in which the placental trophoblast (TB) fails to penetrate the uterine epithelium at all, as in pro-simians (King 1992). This structural heterogeneity presumably reflects evolutionary adaptation to the on-going conflict over control of available nutrient resources, which the mother supplies and the fetus exploits and also the on-going challenge to fetal survival in the face of a potentially hostile and capricious maternal immune system.

**Fig. 1:**
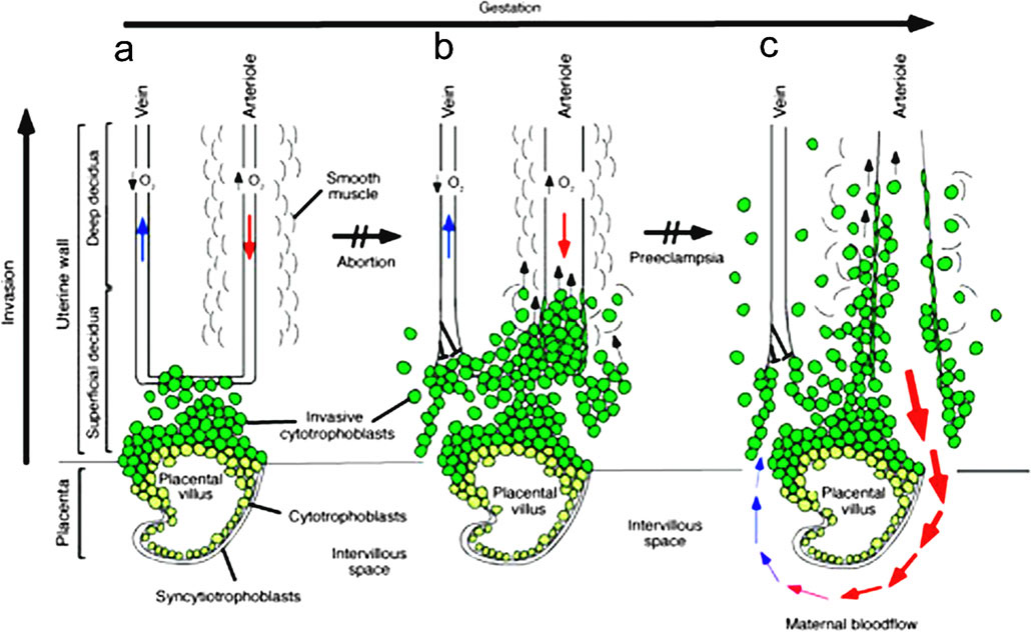
Representation of the human placenta during the process of uterine invasion. After initial penetration into maternal tissues by cells derived from blastocyst trophectoderm, which are believed to form a short-lived syncytium, underlying cytoTBs push through the syncytial mass and form chorionic villi (CV). Early CV form multilayered structures that ultimately give rise to anchoring and “floating” (ones not immediately connected to maternal endometrial tissues) CV. Here, for simplification, only anchoring villi are shown. **a** The early stages of placental development take place in a relatively hypoxic environment that favors cytoTB proliferation rather than differentiation along the invasive pathway. Accordingly, this cell population (*light green cells*) rapidly increases in number as compared with the embryonic lineages. CytoTB also differentiate into syncytioTB, which lines the interface between the placental villous and the intervillous space. This space ultimately becomes filled with maternal blood. **b** As development continues, cytoTBs (*dark green cells*) invade the uterine wall and plug the maternal vessels, a process that helps maintain a state of physiological hypoxia. As indicated by the *short black arrows*, cytoTBs migrate farther up arteries than veins. **c** By 10–12 weeks of human pregnancy, blood flow to the intervillous space begins. As the endovascular component of cytoTB invasion progresses, the cells migrate along the lumina of spiral arterioles, replacing the maternal endothelial lining. CytoTBs are also found in the smooth muscle walls of these vessels. In normal pregnancy, the process whereby placental cells remodel uterine arterioles involves the decidual and inner third of the myometrial portions of these vessels. As a result, the diameter of the arterioles expands to accommodate the dramatic increase in blood flow that is needed to support rapid fetal growth later in pregnancy. It is likely that failed endovascular invasion leads, in some cases, to abortion, whereas an inability to invade to the appropriate depth is associated with preeclampsia and a subset of pregnancies in which the growth of the fetus is restricted. The figure is reproduced from Red-Horse et al. (2004)

The human placenta is morphologically complex (Fig. 1) but does bear some resemblance to that of the mouse (Pijnenborg et al. 1981; Aubuchon et al. 2011). It is discoid in shape, possesses a multinucleated syncytium in association with its absorptive interface and is in direct contact with maternal blood, thereby providing so-called hemochorial exchange for gases, nutrients and placental hormones. There are also some possible functional homologies between the tissue components of the human and mouse placentae, a fortunate circumstance, because mouse genetics have been able to provide useful, though limited, inferences to be drawn about human placental development and pathologies (Cross 2005; Senner and Hemberger 2010). However, such models and comparative morphological and functional studies can only go so far and there continues to be profound ignorance about the early stages of human pregnancy, especially associated with the events accompanying implantation, when embryonic losses are high (Macklon et al. 2002) and about the mechanisms that underpin the emergence of various mature cell lineages from the initiating trophectoderm and its descendants. Ethical and legal considerations, in any case, place curbs on experiments that can be performed on cultured human conceptuses.

In addition to the difficulties in addressing events of early pregnancy, there is also little known about the etiology of placental diseases, such as preeclampsia, which have their origins early but are manifested in the third trimester and most often in the last few weeks of gestation, when fetal demands place considerable stress on the mother even when she is bearing a normal placenta (Roberts and Gammill 2005). As has been pointed out by us (Schulz et al. 2008) and others (Golos et al. 2006; Douglas et al. 2009; Marchand et al. 2011), there remains a need for in vitro systems that can mimic early events in the development of the human placenta better than is possible with immortalized or cancerous TB cell lines and primary cultures of placenta, whether collected at term or from early pregnancy terminations. Importantly, TB stem cells that might be used to track such early events have yet to be isolated from the human, despite success in the mouse (Roberts and Fisher 2011).

As pointed out above, models useful for studying human TB development are limited. TB isolated from term placenta (Frank et al. 2001) has been used to elucidate some aspects of sycytioTB formation (Aplin et al. 2006) but only primary cells from first trimester tissue will spontaneously form extravillous trophoblast (EVT) (Genbacev and Miller 2000). Such material is not widely available and even the primary cells from such sources have only a limited capacity to proliferate and must be repeatedly isolated (Douglas et al. 2009). However, the recent establishment of continuously proliferating cell lines from first trimester chorion with the properties of multipotent cytoTB precursors may remedy this situation (Genbacev et al. 2011).

Choriocarcinoma cell lines display some properties of EVT (King et al. 2000; Shiverick et al. 2001). These lines are easy to maintain and respond to treatments that drive differentiation and invasion but their behavior is often quite different from that of the primary TB they are intended to mimic (Graham et al. 1994; Hohn et al. 1998; Zhang et al. 2002) and their exact place in the TB lineage is unclear. Recent work based on HLA expression suggests that JEG3 cells to some extent resemble EVT, while JAr is more representative of villous TB (Apps et al. 2009). Some anomalies observed in such cells may also be due to their tumor cell origins (Khoo et al. 1998a, b; Lee et al. 2001; Lala et al. 2002). Immortalized cell lines with EVT properties such as TEV-1 (Feng et al. 2005), Swan-71 (Fest et al. 2008), TCL1 (Fukushima et al. 2003), SGHPL-4 (Cartwright et al. 1999) and HTR8 (HTR-8/SVneo) (Graham et al. 1993) are also available. The latter cell line, in particular, contains a subpopulation of cells with multi-lineage differentiation capability (Takao et al. 2010) but it is unclear whether these represent TB stem cells or even their early progeny. Clearly, models are required for studying TB emergence and differentiation, which are easily maintained in culture, yet closely represent normal TB in its early commitment stages.

Two such model systems have surfaced during the past decade to study human TB differentiation from pluripotent precursor cells. The first (“TB from embryoid bodies”), which will be reviewed only briefly, has been to isolate TB from embryoid bodies. The second (“TB derivation through exposure of “primed” pluripotent stem cells to BMP4”), which is the main focus of this paper, is the direct conversion of pluripotent stem cells of human origin embryonic stem cells (hESC) and induced pluripotent stem cells (hiPSC) to cells of the TB lineage by blocking the signaling systems essential for maintaining pluripotency and exploiting instead a competing SMAD1/5/8 pathway through addition of bone morphogenetic protein-4 (BMP4) or related growth factors to the culture medium.

## TB from embryoid bodies

The derivation of TB from embryoid bodies assembled from hESC was described by Gerami-Naini et al. ( 2004) and has been reviewed several times recently (Golos et al. 2006, 2010; Douglas et al. 2009). The approach has been to disperse the cells into clumps, which are then cultured under conditions where they cannot re-adhere. The aggregates are then put under conditions that promote differentiation, usually by replacing the standard ESC culture medium containing FGF2 with one supplemented with fetal bovine serum (FBS). After about a week of culture, the embryoid bodies are allowed to attach to a substratum, usually Matrigel, upon which they form outgrowths enriched in TB. The value of this system is that, to some extent, it appears to mimic the early spatial disposition events of TB formation in the conceptus, with TB enclosing other lineages in a three-dimensional format. It is also possible to reconstitute embryoid bodies with a mixture of cell types, thereby allowing tissue interactions on TB properties to be examined (Giakoumopoulos et al. 2010). The embryoid bodies produce copious amounts of hCG and display a range of diagnostic TB markers. Component cells with a TB phenotype also exhibit migratory properties (Harun et al. 2006; Golos et al. 2010; Udayashankar et al. 2010). Finally, individual cell lines have been derived from such outgrowths by using hCG production as a marker for selection (Harun et al. 2006) but the claim that such cells are stem cells (Harun et al. 2006; Frost et al. 2010) may be overstated in view of the fact that high hCG production is a feature of differentiated TB and a TB stem cell phenotype remains unproven.

## TB derivation through exposure of “primed” pluripotent stem cells to BMP4

### Introduction

The experimental system first described by Thomson’s group (Xu et al. 2002; Xu 2006) and later by us (Das et al. 2007; Schulz et al. 2008) and others (Besser 2004; Gerami-Naini et al. 2004; Golos et al. 2006; Zhang et al. 2007; Wu et al. 2008; Douglas et al. 2009; Erb et al. 2011; Marchand et al. 2011), in which hESC are driven towards TB in response to the growth factor BMP4, is particularly attractive in that it allows the “birth” of TB to be followed from pluripotent progenitors and the subsequent differentiation of these early TB to be followed along specific sub-lineages. This phenomenon, which is observed with primed type ESC, such as those derived from human blastocysts but not in naïve type ESC represented, for example, by mouse ESC (see “Naïve versus “primed”/epiblast-type embryonic stem cells”), can probably be attributed in part to the underlying differences in pluripotency that distinguish the two cell types. In the review that follows, we briefly contrast the naïve and primed pluripotency states and the potential value of using iPSC of the primed type rather than ESC as the TB progenitor cells for species where ESC are unavailable (see “Induced pluripotent stem cells (iPSC) as an alternative to ESC”). Emphasis is given to delineate the underpinnings of the BMP4/hESC model system, including some of its weaknesses and strengths (see “The opposing roles of FGF2 and BMP4 in directing TB lineage formation”). We also describe the likely involvement of O_2_ in the differentiation process (in “Oxygen acceleration of TB differentiation”) and the possibility of employing pharmacological agents rather than just BMP4 to provide directionality to the emergence of TB sub-lineages (in “Drug-driven differentiation of hESC to TB”). Finally, we discuss recent challenges to the model and its legitimacy as an experimental paradigm for TB investigation (in “Do hESC offer a means of generating human TB stem cells?” and “Is TB derived from BMP4-treated hESC and iPSC a mesoderm derivative?”)

### Naïve versus “primed”/epiblast-type embryonic stem cells

Human ESC, despite being derived from the ICM of blastocysts, show little obvious resemblance to mouse ESC, which were first generated almost 20 years earlier, also from blastocysts. Human ESC colonies have a flattened rather than raised morphology and require a different set of growth factors than their mouse counterparts, which depend upon LIF/STAT3 signaling for maintenance of their pluripotency (Hall et al. 2009). These and other differences have been reviewed in several publications and will not be covered in detail here. More recently, a different kind of mouse ESC was derived from the epiblast of gastrulation stage mouse embryos (Brons et al. 2007; Tesar et al. 2007). These cells shared striking similarities to hESC in colony morphology, requirement for activin A and FGF2 and lack of dependence on LIF and have been called “primed” or epiblast stem cells (EpiSC) to distinguish them from the “naïve”, ICM-derived ESC (Nichols and Smith 2009; Hanna et al. 2010b). The mouse EpiSC also differentiate in response to BMP4 in a manner similar to hESC (Brons et al. 2007; Tesar et al. 2007; Vallier et al. 2009; Bernardo et al. 2011). Although EpiSC and naïve ESC can be inter-converted by genetic manipulation and even by selection through altered culture conditions (Bao et al. 2009; Hanna et al. 2010a; Xu et al. 2010), it is clear that the two stemness states are supported by different signaling networks and react differently to directing stimuli, although in reality the naïve and primed states may be in an equilibrium whose dynamic balance can be shifted experimentally (Greber et al. 2010). For example, naïve mESC are prone to TB differentiation in response to BMP4 when they are grown on a laminin or fibronectin surface, possibly because they begin to acquire a primed phenotype on these matrices prior to their differentiation to TB (Hayashi et al. 2010). Considering that naïve ESC are derived from cells that only recently diverged from trophectoderm, it is curious that they resist ready transformation to TB, whereas EpiSC, whose origins are well-separated from the TB lineage readily make the lineage switch. This conundrum is discussed further in “Is TB derived from BMP4-treated hESC and iPSC a mesoderm derivative?” where we consider whether or not EpiSC can be used to provide a source of “true” TB and TB stem cells in light of a recent publication criticizing this model (Bernardo et al. 2011).

### Induced pluripotent stem cells (iPSC) as an alternative to ESC

Reprogramming somatic cells, first with retroviral vectors carrying transcription factor genes characteristic of the stemness state (Takahashi and Yamanaka 2006) and later by other means (Hochedlinger and Plath 2009; Cox and Rizzino 2010), have demonstrated that the pluripotent phenotype can be readily recreated in the laboratory. How similar these cells are to ESC and whether or not they carry an epigenetic memory of their origins continues to be argued (Bock et al. 2011). Nevertheless, iPSC colonies fall into two general phenotypes, ones that resemble naïve, LIF-dependent ESC, e.g. from mouse and those that are clearly of the epiblast type as generated from human and pig somatic cells (Telugu et al. 2011). As far as can be discerned, the epiblast types of iPSC, whether from human or pig, respond to BMP4 in a similar manner as their true EpiSC counterparts and readily form TB (Alberio et al. 2010; Wolfrum et al. 2011). Such cells clearly provide an alternative to using hESC and additionally should allow the transition to TB from iPSC to be compared across species that possess contrasting kinds of specialized placental cell.

### The opposing roles of FGF2 and BMP4 in directing TB lineage formation

In the initial study of Xu et al. (2002), in which BMP4 and several related growth factors, for example BMP2 and BMP7, were used to drive differentiation of several hESC lines, including the now well-studied H1, H7 and H9 cells, to TB, a number of important observations were made. First, differentiation was visible as a progressive gain of epithelial morphology of cells within the colony, a transition that advanced from the periphery towards the center. Second, the rate of colony transformation in terms of acquisition of epithelial phenotype was dose-dependent in relation to the concentration of BMP provided in the medium. At 100 ng, BMP4/ml progression was rapid, whereas at 10 and 1 ng/ml it was significantly slower. Importantly, although not commented upon in detail in the 2002 publication, the absence of FGF2 appeared to enhance BMP4-driven differentiation, an observation later confirmed by number of other investigators (Das et al. 2007; Marchand et al. 2011; Yu et al. 2011). Third, even though BMP2, BMP7 and GDF5 could mimic the effects of BMP4, other members of the TGFB family, including TGFB itself and activin A were ineffective, in retrospect, a not unsurprising observation, as these latter factors, along with FGF2, can help maintain the pluripotent state of hESC. And fourth, there was a progressive up-regulation of gene markers, including ones encoding several transcription factors that are candidate markers for the TB lineage. In addition, many markers of “mature” syncytial TB, such as CGA and CGB, were up-regulated quite late in the process, usually after several days, suggesting that the transformation to TB proceeded through a number of intermediate steps before functionally mature cells appeared. Nonetheless, there was also evidence that the process, at least in its initial stages, was not exclusively to TB, as close examination of the microarray data at early time points reveals there was expression of primitive (yolk sac) and definitive endodermal gene markers, such as *AFP* (alpha-fetoprotein), *RBP4* (retinol binding protein 4) and *FGG* (fibrinogen-γ).

Experiments performed in several laboratories, including our own (Das et al. 2007; Schulz et al. 2008), subsequently confirmed most of the above observations. In our case, we initially concentrated on how FGF2 and oxygen tension played into the differentiation process. Using a single, relatively low BMP4 concentration (10 ng/ml) and a 2 × 2 factorial design, we compared differentiation of H1 and H9 cells in the presence and absence of 4 ng/ml FGF2 and under contrasting gas atmospheres of 20% and 4% O_2_. The results were quite clear. FGF2 slowed the differentiation process under the two gas atmospheres both in terms of morphological transformation of the cells and the production of the hormones hCG and progesterone (P4). Indeed, in the presence of FGF2, P4 release into the medium over a 5-day time course was almost completely suppressed under either O_2_ condition (Das et al. 2007). It was also evident that late differentiation events were additionally slowed under low O_2_ conditions but otherwise appeared to progress not very differently than under high (20%) O_2_. Accordingly, FGF2 was omitted from the culture medium in all our subsequent experiments when BMP4 was employed to drive TB differentiation (Das et al. 2007; Schulz et al. 2008).

The complicating nature of the presence of FGF2 on BMP4 differentiation has been observed by others. For example, the tendency of hESC to differentiate spontaneously during routine culture could at least in part be attributed to the presence of BMPs that antagonized the ability of FGF2 to maintain the pluripotent state, suggesting that the signaling pathways activated by the two growth factors interacted in some manner (Wang et al. 2005; Xu et al. 2005; Ludwig et al. 2006; Lin et al. 2010). Subsequently, Yu et al. (2011) noted that, in a defined culture medium developed specifically for maintaining hESC in a pluripotent state and containing relatively high concentrations of FGF2 (mTeSR medium) (Ludwig et al. 2006), FGF2 signaling through the MEK/ERK signaling pathway negated the effects of BMP4 in promoting TB differentiation. Instead, the combination of the two growth factors resulted in prolonged *NANOG* expression and up-regulation of primitive streak gene markers, especially *T* (brachyury), as well as endoderm and mesoderm marker genes. Only when FGF2 in the medium was reduced in concentration or omitted completely was BMP4 able to induce TB formation efficiently and only in the absence of BMP4 was pluripotency maintained. More recently, Bernardo et al. (2011) have argued that the role of BMP4 is to block endoderm formation and permit differentiation to mesoderm and that the cells expressing TB markers are not “true” TB at all but a derivative of mesoderm arising as an artifact of in vitro culture. Indeed, these workers insist that hESC do not have any capacity to generate TB “simply by addition of BMP4 to their growth medium in either the presence or absence of FGF2”. We attempt to refute this argument later in this paper.

**Fig. 2:**
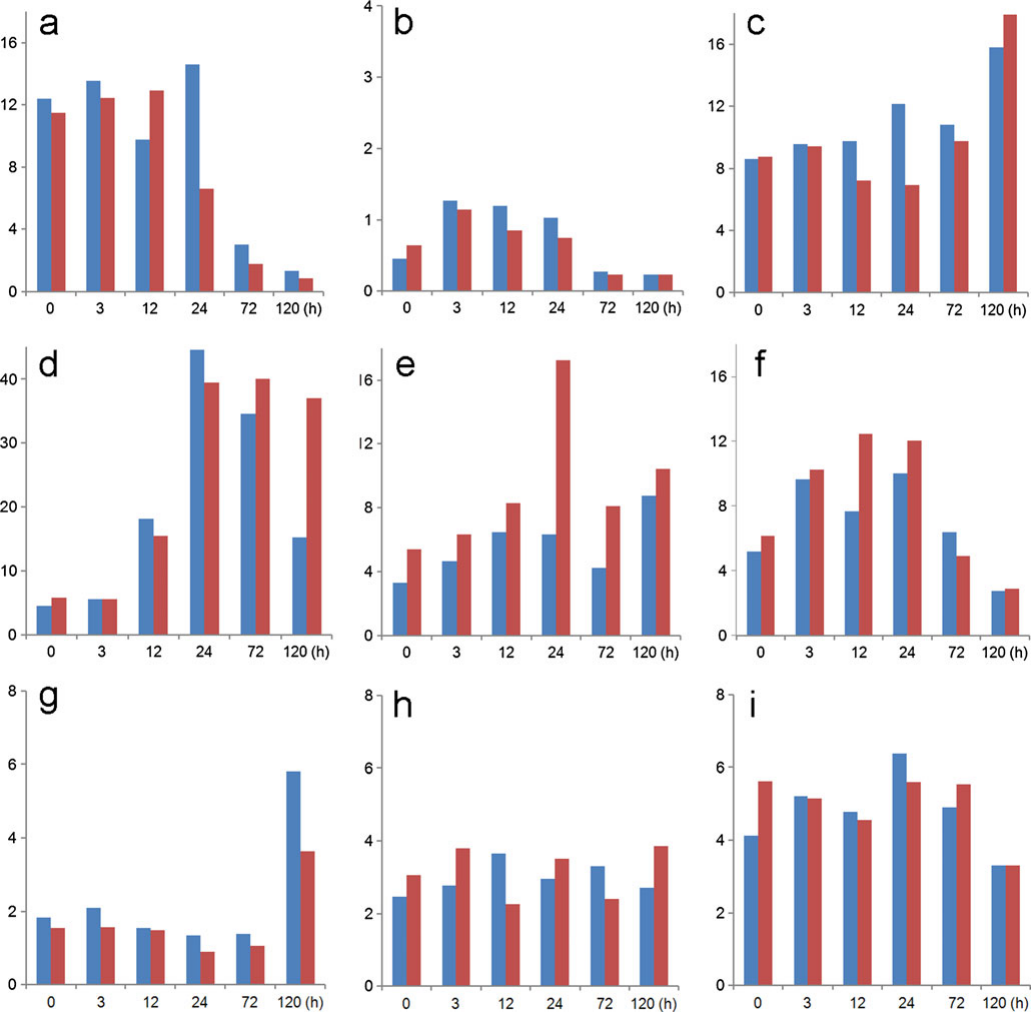
Relative transcript concentrations of several genes for growth factors and their cognate receptors whose expression could influence differentiation events in BMP4-treated H1 cells. Data (GEO GSE10469) were obtained by microarray over time and in response to O_2_. Values were normalized to the median intensity of the array. H1 hESC were supplemented with BMP4 (10 ng/ml) under either 4% (*blue*) or 20% (*red*) O_2_ conditions. RNA isolated at 0, 3, 12, 24, 72 and 120 h (Schulz et al. 2008). These data were obtained on Agilent Whole Human Genome Oligo microarrays. The value represents a normalized expression (1.0 being the signal at the 50th percentile). For details of methods, see Schulz et al. (2008). Values shown are for **a** (*FGF2*), **b** (*FGF4*), **c** (*FGFR2*), **d** (*BMP4*), **e** (*BMP7*), **f** [*BMPR1A* (*ALK3*)], **g** (*INHBA*) (gene encoding activin A subunits), **h** [*ACVR1* (*ALK2*)] and **i** [*TGFBR1* (*ALK5*)]. Note that the cells may continue to have the potential to respond to endogenously produced FGF2 and activin A even when exogenous sources are absent

The complex nature of the BMP4/FGF2 relationship is also emphasized when the expression patterns of their genes are analyzed. H1 hESC maintained on a medium conditioned by MEF, which likely contains BMP4 (Wang et al. 2005; Xu et al. 2005; Lin et al. 2010; Yu et al. 2011), express both *FGF2*, *BMP4* and *INHBA* (gene encoding activin A subunits) (Fig. 2), which may contribute to the unstable phenotype of the cells, i.e., spontaneous differentiation (Ezashi et al. 2005; Westfall et al. 2008). Upon exposure to added BMP4, expression of *FGF2* (Fig. 2) and genes encoding its regulators, *POU5F1* and *SOX2* (Schulz et al. 2008), fall, while transcript concentrations for *FGFR2*, whose two main splice variant forms bind a variety of FGF ligands, including FGF2 and FGF4 (Katoh and Katoh 2009), remain relatively unchanged (Fig. 2). Additionally, there is minor up-regulation of FGF4, a growth factor essential for maintaining the self-renewal of TB stem cells in the mouse (Tanaka et al. 1998) and a product of the pluripotent ICM and epiblast of mouse embryos, where it probably acts locally to promote TB proliferation. The expression of the genes encoding BMP4 and its major cognate receptor, *BMPR1A* (*ALK3*), rises after application of BMP4 to H1 cells (Fig. 2), thereby ensuring a positive feed-back loop. Transcripts for *BMP7* are also detectable in the H1 cells both before and after BMP4 exposure, so that its protein product may reinforce the conditions promoting ESC differentiation. It is also important to note that genes encoding activin A (*INHBA*) and its cognate receptors are also maintained in the H1 cells after BMP4 exposure (Fig. 2), raising the possibility for conflict between the pathways driving differentiation with those that maintain pluripotency.

The fact that added FGF2 opposes the directionality of BMP4-driven differentiation of hESC towards TB has meant that most researchers seeking to follow TB emergence no longer include FGF2 in the medium after BMP4 is added. This practice has the additional advantage of allowing the use of lower BMP4 concentrations to provide relatively rapid transformation of the colonies to TB and a significant cost savings to the investigator. In addition, exclusion of FGF2 avoids the diversion of the hESC to lineages other than TB quite efficiently. For example, microarray analysis of H1 cells after 3, 12, 24, 72 and 120 h treatment with 10 ng/ml BMP4 (GEO GSE10469) revealed immediate up-regulation of transcription factors associated with TB emergence, e.g. *CDX2, ID2, LEF, EBF3, HAND1, DLX3* and *5, MSX2, GATA2* and *3*, *FOXF2*, with no evidence for up-regulation of expression of lineage markers for mesoderm (*WNT3A*, *BRACHYURY/T*), endoderm (*HOXB1, DPF3*) and ectoderm (*PAX6, ZIC1*) over the time course of treatment (GEO GSE10469). Thus, predominantly TB emerges when hESC are treated only with BMP4. These observations were subsequently confirmed by Marchand et al. (2011).

### Oxygen acceleration of TB differentiation

The human conceptus, like that of other species, initially develops under conditions that are approaching hypoxic and certainly low in O_2_. As ESC are derived from the ICM or epiblast of such conceptuses, we hypothesized that such cells could be grown successfully under such hypoxic conditions. This hypothesis proved to be correct. H1 cells, for example, divided just as quickly under 3 and 5% O_2_ as they did under 20% O_2_ and were only slowed slightly at 1% O_2_ (Ezashi et al. 2005). On the other hand, TB cells, which initially surround the inner pluripotent cells, seem likely to be exposed to somewhat higher O_2_ conditions than inner cells from the time the lineages separate and may mop up much of the available O_2_. Moreover, towards the end of the first trimester of human pregnancies, the intervillous space becomes fully perfused with oxygenated maternal blood (Burton et al. 2002). Therefore, TB cells on or near the surface of the fetal villi will face O_2_-rich conditions. Similarly, the extravillous TB component of the placenta invading into the uterine wall will also be exposed to increasingly higher O_2_ tensions as it approaches maternal arterioles. Such conditions reduce cellular proliferation and favor differentiation of cytoTBs (Huppertz et al. 2009). Thus, it seemed reasonable to include O_2_ as a variable in studies that employ the BMP4/hESC model.

As described earlier, differentiation is accelerated under a high O_2_ atmosphere in the sense that the colonies make the transition to an epithelial morphology more rapidly than at low O_2_ (Das et al. 2007). Another longer term morphological feature of BMP-treated hESC colonies is the formation of syncytial TB, a cell type that occupies extensive areas of the colonies under high O_2_ atmosphere, appearing 72–120 h after BMP4 exposure but more slowly in colonies maintained under 4% O_2_. Gene markers of syncytioTB reflect these differences in that their appearance is delayed by approximately 24 h (Schulz et al. 2008). Clearly, O_2_ has a profound influence on the rate at which BMP4 drives hESC to terminally differentiated TB.

**Fig. 3:**
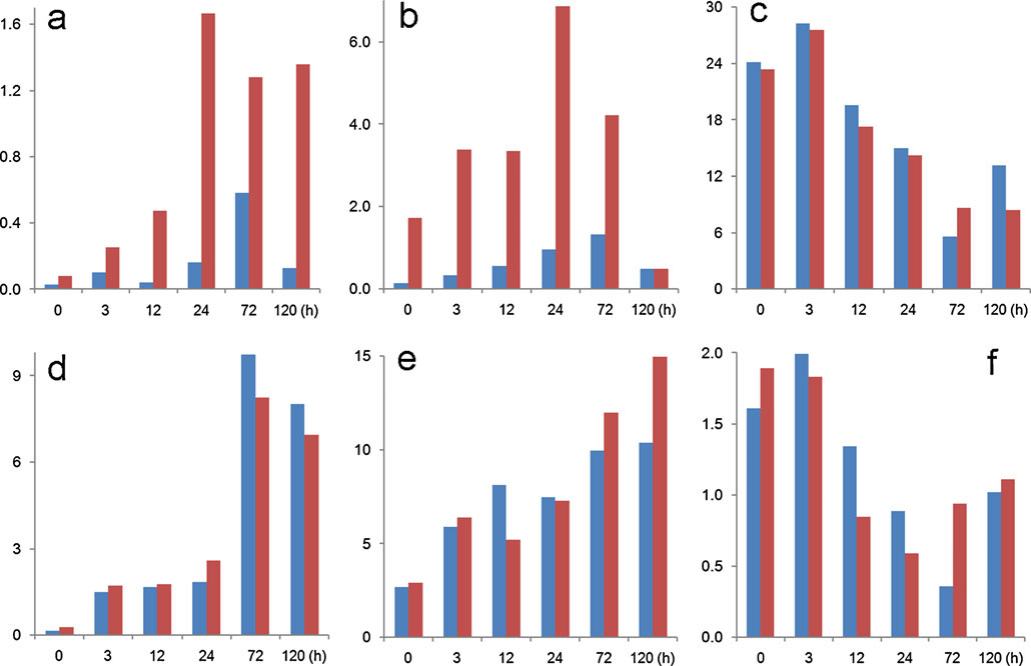
Relative transcript concentrations of genes encoding transcription factors that have been implicated in TB lineage emergence (Roberts and Fisher 2011). Values are shown for **a** (*CDX2*), **b** (*EOMES*), **c** (*ETS2*), **d** (*GATA3*), **e** (*TFAP2C*) and **f** (*ELF5*). The data were obtained as described in the legend to Fig. 2 from cells maintained under either 4% (*blue*) or 20% (*red*) O_2_ conditions. RNA was isolated at 0, 3, 12, 24, 72 and 120 h after BMP4 exposure (GEO GSE10469)

The initial, BMP4-controlled, up-regulation of several transcription factors believed to be associated with TB stem cells and TB emergence, discussed in “The opposing roles of FGF2 and BMP4 in directing TB lineage formation”, is also oxygen sensitive. Such O_2_ response differences are particularly obvious for *CDX2* and *EOMES* (data that have been confirmed by quantitative RT-PCR; not shown), for example, whereas others, e.g. *ETS2* and *GATA3*, seem less influenced by the gas atmosphere (Fig. 3), the basis of these differences in relation to O_2_ tension is unclear but are presumed related to the relative instability of the HIF1A transcription factor as O_2_ tensions rise (Semenza 2007). The data are also consistent with previous observations from our laboratory that under 20% O_2_ the cells are more poised to differentiate than when they are under the low O_2_ conditions (Westfall et al. 2008). Key controllers of these events may be LEFTY1 and 2, whose genes display a 2- to 3-fold higher expression under 4% as compared to 20% O_2_ in hESC and are rapidly down-regulated either by addition of BMP4 (Schulz et al. 2008) or by inhibiting the activin A-linked kinases ACVR1B, TGFBR1 and ACVR1C with the drug SB431542 (Besser 2004). LEFTY proteins, which are members of the TGFB superfamily, are regulated in pluripotent stem cells by a combination of SOX2 and POU5F1 (Boyer et al. 2005). LEFTY2, in particular, appears to antagonize differentiation initiated by NODAL signaling by blocking the formation of an active NODAL/activin A receptor complex (Schier and Shen 2000; Sakuma et al. 2002; Schier 2003). NODAL is a required factor for mouse TB stem cell renewal and, as a product of mitotically inactivated mouse embryonic fibroblasts, has likely been present in most culture media used to support hESC until the recent availability of defined media (Roberts and Fisher 2011). Again, such data support the idea that the pluripotent networks supporting stemness are relatively unstable and are further compromised as O_2_ tensions rise.

### Drug-driven differentiation of hESC to TB

Self-renewal and conservation of pluripotency in hESC requires either activin A or related factors (Beattie et al. 2005; James et al. 2005; Vallier et al. 2005; Chng et al. 2011) and FGF2 (Vallier et al. 2005; Wang et al. 2005). As pointed out above, the former binds to the ACVR1B, TGFBR1 and ACVR1C receptors, whose ability to initiate downstream signaling events, namely activation of the transcriptional modulators SMAD2 and SMAD3, can be blocked by the kinase inhibitor 4-[4-(1,3-benzodioxol-5-yl)-5-(2-pyridinyl)-1H-imidazol-2-yl]benzamide, better known as SB431542 (Laping et al. 2002). Hence, SB431542 has become widely used to block TGFB/activin/NODAL signaling in a variety of cell types. One outcome of such inhibition of activin A signaling in hESC is to interfere with the transcription of key “stemness” genes, such as *NANOG* (Xu et al. 2008; Vallier et al. 2009) and *LEFTY1* and *2* (Besser 2004) and consequently to impede the signaling network that sustains hESC self-renewal. As a consequence, SB431542 can be used in conjunction with BMP4 to enhance TB emergence from hESC (Wu et al. 2008; Erb et al. 2011) and provides a cheaper alternative to using natural polypeptide inhibitors such as follistatin (Vallier et al. 2005; Saha et al. 2008). More specific and more potent inhibitors of activin A signaling, such as A83-01 (Tojo et al. 2005; Garamszegi and Garamszegi 2011), may provide improved tools for directing hESC to TB (see ‘‘Is TB derived from BMP4-treated hESC and iPSC a mesoderm derivative?”).

FGF2 is a routine component of both complex and defined media used to culture hESC but exactly how this growth factor, which is active only as a complex with heparan sulfate, enhances self-renewal, is still not completely understood, in large part, because of the complexities that arise in studying the various spliced forms of FGF receptors, which differ from each other in ligand-binding affinities, tissue distribution and differential coupling to distinct intracellular signaling pathways (Eswarakumar et al. 2005). What is clear is that when FGF2 binds to one or more of its cognate receptors, each of which possess a tyrosine kinase domain within their intracellular domains, there is a cascade of phosphorylation events involving several signaling pathways (Ding et al. 2011; Zoumaro-Djayoon et al. 2011). The one that appears to be most relevant to the maintenance of stem cell renewal and pluripotency involves RAS/RAF/MEK1/2 and leads to activation of ERK1/2 (Kang et al. 2005; Li et al. 2007) and, as with activin A signaling, maintenance of NANOG levels (Yu et al. 2011). The availability of a wide range of inhibitors, with varying affinities for the protein kinases components of FGF receptors and their downstream targets (Katoh and Katoh 2009) provides additional potential tools for directing TB differentiation from hESC. For example, PD173074 has already been employed to counteract the tendency of FGF2 to direct differentiation of hESC to endoderm rather than TB in presence of BMP4 (Yu et al. 2011). Complete blockade of the FGF2 and activin A response pathways in hESC, will undoubtedly enhance our ability to guide hESC and iPSC to TB more uniformly (see “Is TB derived from BMP4-treated hESC and iPSC a mesoderm derivative?”).

Pharmacological approaches to directing the fate of stem cells along particular lineages has major advantages to the investigator, not the least of which is the stability and consistency of such reagents relative to polypeptides, such as BMP4, which we have noted to exhibit disconcerting differences in potencies from lot-to-lot and between different suppliers. Indeed, some of the inconsistencies observed between investigators in BMP/hESC experiments may be due to differences in BMP4 quality, density and properties of the feeder cultures providing supporting growth factors and the size and density of the hESC colonies after they are sub-cultured. Combinations of FGFR and activin A receptor inhibitors in concert with defined culture media and careful quality control over passaging conditions and the progression of differentiation across the colonies are likely to provide a simpler and more uniform approach to examining TB emergence from hESC.

### Do hESC offer a means of generating human TB stem cells?

Mouse TB stem cells were first established from outgrowths of trophectoderm and explants of extra-embryonic ectoderm dissected from the base of the ectoplacental cone bordering epiblast over 13 years ago (Tanaka et al. 1998), although it is not completely clear whether the two types of TB stem cell so generated are equivalent. Similar approaches had not yielded comparable types of cells from a primate species until they were generated from Rhesus monkey blastocysts (Vandevoort et al. 2007) but comparable human cells have not been described from blastocyst outgrowths (Genbacev et al. 2011). Although undoubtedly TB, the monkey lines warrant additional characterization, as they display a number of atypical markers and growth features that sets them apart from mouse TB stem cells (Roberts and Fisher 2011). Although clusters of dividing cells are present at the base and tips of cytoTB columns in first trimester placental villi that carry markers, such as ELF5 and CDX2, similar to those found on cultured mouse TB stem cells (Hemberger et al. 2010), such cells have not been isolated and characterized further. The TB-like cells recently isolated from chorionic membranes may, however, be bona fide candidates as TB progenitor cells, even though they again differ in many respects from mouse TB stem cells (Genbacev et al. 2011). Early passage cells were initially positive for POU5F1 and expressed a number of TB markers, including EOMES, GATA3 and GCM1 and can be differentiated into cell types that express hCG and HLAG. On the other hand, the cell lines selected from embryoid bodies on the basis of CGB expression by Harun et al. (2006) seem unlikely to represent TB stem cells, because CGB and its partner CGA are products primarily of syncytioTB and not cytoTB. These genes are not significantly expressed in colonies of BMP4-treated hESC until at least 3 days after initiation of differentiation (Schulz et al. 2008).

BMP4-treated hESC in the early stages of differentiation may provide an opportunity to generate human TB stem cell lines, although there is no certainty that self-renewing multipotent cells exist in the colonies. Moreover, even if they are present, the technical challenges of isolating and propagating the cells will remain. Nevertheless, after BMP4 exposure genes for several transcription factors implicated in establishing mouse TB stem cell self-renewal and multipotency, including CDX2, EOMES, TFAP2C and GATA3, are up-regulated within 3 h, while the mRNA for others, including ELF5, ETS2 and TEAD4, which are also expressed in undifferentiated hESC, remain expressed as the transition proceeds (Fig. 3; and unpublished data available through GEO GSE10469). Such changes occur before KRT7, the characteristic cytokeratin of TB, is expressed and several days before CGA and CGB can be detected (Schulz et al. 2008). The view that the “predominant cell type that emerges from the hESC to TB transdifferentiation” is syncytioTB (Hemberger et al. 2010) is simply a misconception. Even after day 5, syncytioTB is found only at the periphery of the colonies (Das et al. 2007), at a time when most have converted completely to a KRT7-positive phenotype. The time to seek TB stem cells would seem to be in the early, rather than the late, phase of the trans-differentiation process and before the specialized tissue types have appeared.

ELF5 expression in hESC is of particular interest. In the mouse, ELF5 has been proposed to be a “lineage gatekeeper” controlling the transition of pluripotent progenitor cells, where CpG islands within the promoter of the gene are hypermethylated and inactive, into the TB lineage where the promoter is hypomethyated and active (Ng et al. 2008). Even though Hemberger et al. (2010) have reported that the *ELF5* variant (*ELF2b*) expressed in proliferating human TB and TB cell lines is hypomethylated in its promoter and hence active, it was hypermethylated and inferred as silent in several human ESC and iPSC lines. Nonetheless, although the gene is expressed in the H1 and H9 lines used in our laboratory, it is not perceptibly up-regulated after BMP4 treatment, as is one of its presumed downstream targets, *EOMES*. By contrast, *CDX2* is relatively silent in the pluripotent cells but is up-regulated within 3 h after BMP4 exposure (Fig. 3). Clearly, the epigenetic switch postulated to govern TB emergence and to control ELF5-driven expression of *CDX2* and *EOMES* may be more complicated in the BMP4/hESC system than envisaged in the model proposed by Hemberger et al. (Ng et al. 2008; Roper and Hemberger 2009).

### Is TB derived from BMP4-treated hESC and iPSC a mesoderm derivative?

As mentioned earlier, Bernardo et al. (2011) consider that BMP4 treatment of hESC in absence of FGF2 and activin A does not generate TB at all but instead gives rise solely to mesoderm derivatives. The evidence for this is based primarily on the following findings.

**Fig. 4:**
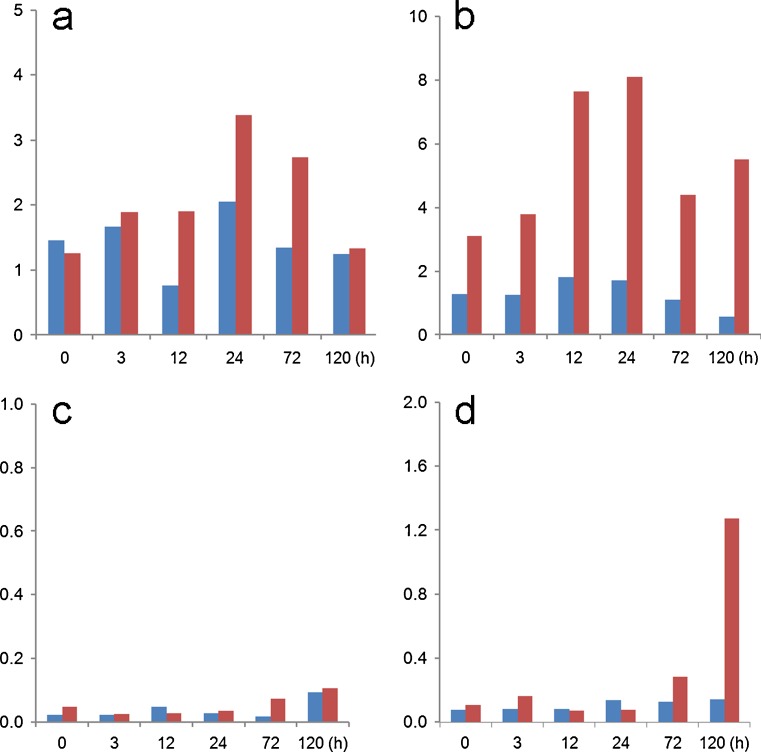
Relative transcript concentrations of genes encoding mesoderm-associated markers: **a** (*T*), **b** (*KDR*), **c** (*VCAM1*) and **d** (*TBX4*) (Bernardo et al. 2011). The data were obtained as described in the legend to Fig. 2 from cells maintained under either 4% (*blue*) or 20% (*red*) O_2_ conditions. RNA was isolated at 0, 3, 12, 24, 72 and 120 h after BMP4 exposure (GEO GSE10469)

**Fig. 5:**
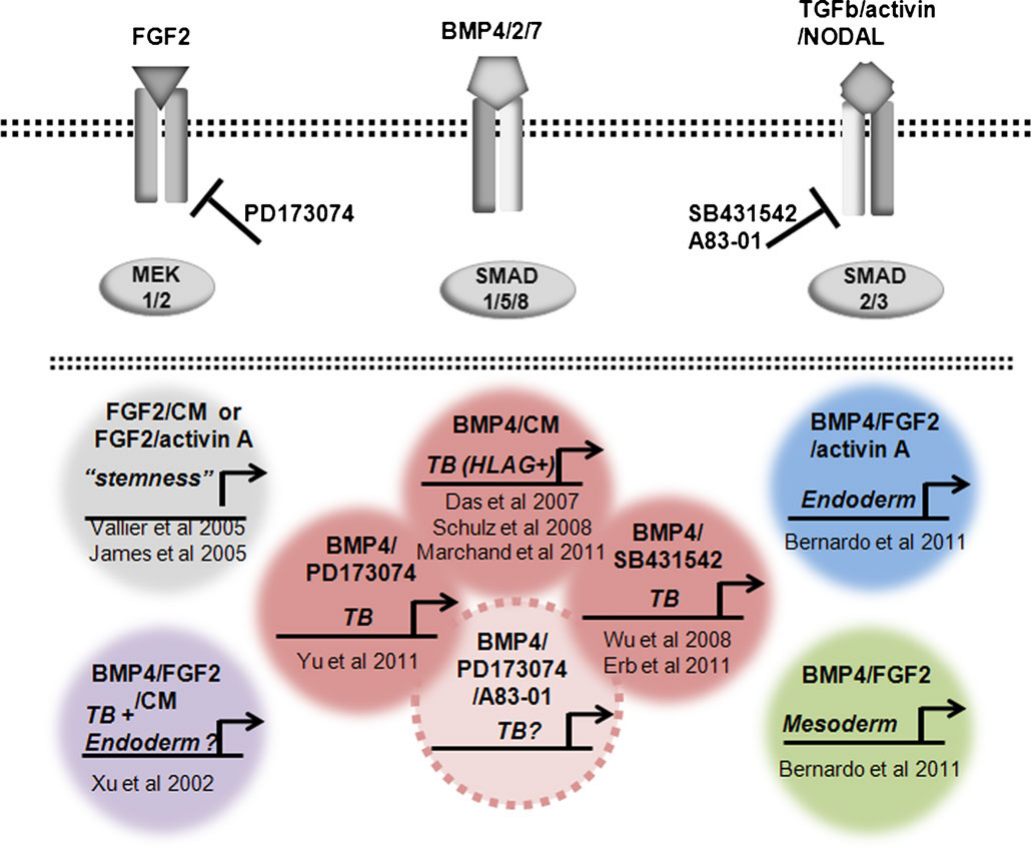
The opposing roles of FGF2 and BMP4 in directing TB formation from epiblast type ES cells and iPS cells and the need to exclude FGF2 from the growth medium to minimize the co-induction of mesoderm and endoderm. Also indicated are the potential values of including low molecular weight pharmaceutical agents that block activin A (INHBA) and FGF2 signaling to support BMP4-directed differentiation to TB. Whereas FGF2 and conditioned medium (CM) from mouse embryonic fibroblasts, which contains activin A and other growth factors, enable maintenance of the pluripotent state of epiESC (*top left*), adding high concentrations of BMP4 leads to TB differentiation and possibly also some endoderm and mesoderm differentiation (*bottom left*). We argue that when BMP4 is present with FGF2 the extent of emergence of TB will depend on the relative concentrations of the two growth factors. By contrast, a defined medium supplemented with BMP4, FGF2 and activin A appears mainly to generate endoderm (*upper right*). However, the same defined medium formulated for minimal activin A signaling gives rise predominantly to mesoderm (*lower right*). Addition of BMP4 but minimizing FGF2 contribution to signaling either by excluding it from the culture medium or blocking MEK1/2 signaling, strongly favors TB formation, as does the inhibition of the activin A-mediated SMAD2/3 pathway (*triad cluster in center*). Blocking signaling through both the SMAD2/3 and MEK1/2 pathways in the presence of BMP4 will most likely provide optimal, unidirectional differentiation towards TB and the HLAG+ /cytoTB and CG+/syncytioTB sublineages (*dashed circle in center*)

First, both BMP4 and the activin A signaling inhibitor SB431542 appeared to up-regulate the brachyury gene (*T*), a transcription factor that during embryo development is first expressed in mesoendoderm, the immediate precursor of mesoderm and endoderm, around the time of blastulation (Jones et al. 1991; Winnier et al. 1995). However, close examination of the immunoblots shown in fig. 1B of the paper by Bernardo et al. (2011) indicates little or no effect of BMP4 on brachyury protein concentrations in H9 cells when compared to untreated control cells. Moreover, our microarray data also indicate that the *T* gene is not immediately responsive to BMP4 (Fig. 4) and that its transcripts are present in initiating H1 cells even before their exposure to BMP4. This expression of *T* in hESC and in the presumed TB cells generated by BMP-treatment of hESC is not surprising in view of the fact that brachyury protein is expressed, as assessed by western blotting, both in pluripotent teratocarcinoma cells and in at least two TB cell lines, including JAr (Gokhale et al. 2000). Clearly, this marker may not be a faithful indicator of emerging mesodermal differentiation. One final point with regard to brachyury is worth noting. When Bernardo et al. (2011) up-regulated *T* and consequently mesoderm specification by exposing H9 cells to BMP4 in presence of FGF2, this response was completely abolished when endogenous FGF signaling was inhibited with the inclusion of FGFR or ERK signaling pathway inhibitors (fig. 1C in Bernardo et al. 2011) (Yu et al. 2011). These outcomes are not consistent with an ability of BMP4 alone to regulate *T*. Thus, it would seem essential to eliminate endogenous as well as exogenous FGF signaling in hESC to prevent any residual mesoderm specification occurring in response to BMP4. Accordingly, a useful approach to ensuring that only TB emerges in response to BMP4 will be to block both activin A and FGF signaling simultaneously by including either SB431542 or a more specific ALK inhibitor, such as A83-01 (see “Drug-driven differentiation of hESC to TB”) (Fig. 5).

Second, according to Bernardo et al. (2011), three genes with essential roles in embryonic and extra-embryonic endoderm, namely *KDR* (*FLK1*), *VCAM1* and *TBX4*, were up-regulated by BMP4 at 36 h in the H9 hESC line. Our data (Fig. 4), however, indicate that with the possible exception of *KDR/FLK1*, a member of the VGF receptor family and known to be expressed in human EVT (Iacob et al. 2008), these genes are expressed relatively weakly in H1 hESC, with very little up-regulation by BMP4 over the initial 3 days of exposure. In any case, fold up-regulation parameters can be very misleading when expression in the initiating control cells hovers close to baseline, e.g., *VCAM1 and TBX4*.

Third, *CDX2*, often considered a marker of emerging TB, was more strongly up-regulated in presence of FGF2 plus BMP4 than when FGF2 was omitted (fig. 1A in Bernardo et al. 2011), suggesting that this transcription factor is involved in mesoderm as well as TB specification. Moreover, when *CDX2* was silenced, there was a corresponding fall in expression of mesodermal markers. The results are consistent with CDX2 expression correlating with and possibly playing a role in mesoderm emergence but they certainly do not rule out its involvement in specification of TB when FGF2 is absent from the medium.

Fourth, the ELF5 gene remained hypermethylated in BMP4-treated hESC and only a small percentage of the cells expressed the protein. Since *ELF5* promoter hypomethylation is a feature of mouse TB stem cell, it is argued that the BMP-exposed cells could not be TB. However, the methylation status of the *ELF5* promoter in the minority of the cells that actually expressed ELF5 was not determined and it seems conceivable that this sub-population might represent early stage, emerging TB within the colonies.

Fifth, whereas the BMP4-treated hESC expressed surface class I HLA molecules (for the most part HLA-A and HLA-B), they did not express the non-polymorphic HLA-G and -C molecules (fig. 4C in Bernardo et al. 2011). The absence of HLA-G is particularly puzzling, as, in our hands, H1 colonies treated with 10 ng/mlBMP4 and interrogated by use of a well-defined monoclonal antibody (McMaster et al. 1995) as a detection agent exhibited strong signals for both surface and internal HLA-G (Das et al. 2007). Moreover, such colonies also express HLA-G transcripts (GEO GSE10469), an outcome independently described by others (Marchand et al. 2011). It is difficult to reconcile these contrasting data and interpretations until the experiments are repeated with additional cell lines, events followed carefully over time after BMP4 exposure and consideration being given to the fact that markers such as *T* and *KDR*/*FLK1*, may not be absolute in their specificities.

An essentially theoretical line of reason has also been raised against the BMP4/hESC model for studying TB differentiation, namely that hESC are generated from the inner cell mass of expanded blastocysts when an ICM contribution to trophectoderm is unlikely to occur. In the case of mouse EpiSC, the lines are created from an even more advanced stage of development, the late epiblast. This argument is difficult to counter, although it would be naïve to assume that any of the pluripotent cells are exact equivalents of their progenitors. Instead, they may be derived from only a subpopulation within the tissue of origin or even artifacts of in vitro culture.

A further argument against BMP4 specification of TB is that mouse embryos, in which both copies of the BMP4 gene have been inactivated, implant successfully and on time and only fail in their development at the egg cylinder stage (Winnier et al. 1995), a phenotype consistent with a role for BMP4 in gastrulation and mesoderm formation but not in establishment of TB and implantation. On the other hand, Winnier et al. (1995) also noted that there was partial rescue of a significant number of the homozygous mutants, i.e. some survived to term and beyond, possibly through action of either “related proteins” or through complementation by maternal BMP4. In this regard, mRNA for multiple BMP genes, including those for BMP4, BMP2 and BMP7, are strongly expressed in decidual tissue on the mesometrial side of the uterus adjoining implantation sites. BMP7 may be of particular relevance in this regard. First, like BMP4, BMP7 supports hESC conversion to TB (Xu et al. 2002); second, it is expressed in uterine epithelium of the mouse (Ozkaynak et al. 1997) and in the primary decidual zone in contact with the ectoplacental cone during implantation (Ying and Zhao 2000). Conceivably, one or more maternal BMP proteins and possibly BMP7 rather than BMP4, provide the stimulus for trophectoderm outgrowth and establishment of a rudimentary placenta, both in these mutants and in wild-type conceptuses.

In our view, the line of reasoning provided by Bernardo et al. (2011) that TB is not formed when hESC are exposed to BMP4 is flawed, although we do not quarrel with the possibility that TB might carry a partial mesoderm identity. Based on the available evidence, we suggest that, when signaling networks supporting pluripotency in epiblast-type ESC or iPSC (namely the FGF and the activin A signaling axes mediated through FGF2/ERK and SMAD2/3, respectively) become unsustainable and when specification towards extra-embryonic mesoderm and endoderm are simultaneously blocked, TB emerges as a major default state to pluripotency (Ezashi et al. 2011; Roberts and Fisher 2011) (Fig. 5). The inclusion of BMP4 under these conditions is probably necessary both to accelerate the process and to prevent the acquisition of a neuronal phenotype (Avery et al. 2010) as an alternative to TB.

## Concluding remarks

The weight of evidence indicates that BMP4 can convert hESC and iPSC unidirectionally to TB, provided that the signaling pathways supporting pluripotency or driving endoderm and mesoderm specification are rendered inoperative (Fig. 5). The model has the promise to provide a series of snapshots into the spatial and temporal changes that accompany initial differentiation from pluripotent cells and the subsequent formation of sycytialTB and other sublineages as they arise from cytoTB precursors. It may also afford insight into the molecular events that trigger these developmental transitions. The model should be adaptable to a three-dimensional format and to studying the interaction between TB and other cell types, including blood vessels.
